# Natural Products and Health Care Functions of *Inonotus obliquus*

**DOI:** 10.3390/cimb47040269

**Published:** 2025-04-10

**Authors:** Yiming Wang, Jingsheng Gu, Jiaying Wu, Yuxuan Xu, Yiting Liu, Fengxiu Li, Qiao Liu, Kailun Lu, Ting Liang, Jingwen Hao, Ludan Li, Xiaoying Cao, Jihong Jiang

**Affiliations:** The Key Laboratory of Biotechnology for Medicinal and Edible Plant Resources of Jiangsu Province, School of Life Sciences, Jiangsu Normal University, Xuzhou 221116, China; 1020210013@jsnu.edu.cn (Y.W.); gujingsheng@jsnu.edu.cn (J.G.); 1020220016@jsnu.edu.cn (J.W.); a1015858490@163.com (Y.X.); m19851605508@163.com (Y.L.); 2020241758@jsnu.edu.cn (F.L.); liu_qiao014@jsnu.edu.cn (Q.L.); 1020220017@jsnu.edu.cn (K.L.); lt213@jsnu.edu.cn (T.L.); haojingwen2018@163.com (J.H.); lild@jsnu.edu.cn (L.L.); cxy4868@jsnu.edu.cn (X.C.)

**Keywords:** *Inonotus obliquus*, natural products, pharmacological effects, chaga

## Abstract

With the increasing attention of modern medicine to natural medicinal agents, *Inonotus obliquus* (chaga), a macrofungus with remarkable medicinal value, has gradually garnered widespread academic interest. This paper reviews the primary bioactive components of *I. obliquus* in recent years, including polysaccharides, phenolic compounds, and triterpenoids, which exhibit diverse pharmacological effects such as antioxidant, anti-inflammatory, immunomodulatory, and antitumor activities. It further discusses how these bioactive components enhance human health and disease resistance through distinct biological mechanisms, such as the activation of antioxidant systems, regulation of immune responses, and modulation of apoptosis pathways. Additionally, the article explores the biosynthetic pathways of *I. obliquus* metabolites and their pharmacological relevance. Finally, we summarize the potential of *I. obliquus* as a natural medicinal resource and envisage its future applications in clinical drug development. This review aims to provide novel perspectives for the cultivation, utilization, and industrial-scale exploitation of *I. obliquus*.

## 1. Introduction

In the 16th and 17th centuries, the medicinal properties of *Inonotus obliquus* were recognized in Russia and Northern Europe, and it was used as a folk medicine for cleaning, disinfection, and the treatment of stomach diseases [1,2,3]. In addition, it has no unacceptable toxic and side effects [4]. It is a fungus of the family *Hymenochaetaceae* of the phylum *Basidiomycota*, mainly parasitic on the trunk of birch trees in cold areas of the northern hemisphere [1,5]. Due to the abundance of melanin, most of the sclerotia and mycelium are black [5]. It is also called chaga in Russia because of its irregularly formed sterile conk with burnt charcoal on the outside [1]. *I. obliquus* grows slowly (1–2 cm per year), which means that *I. obliquus* with a diameter of more than 10 cm must have been growing for at least 10–15 years [6,7]. Because of these reasons, the exploration and effective use of *I. obliquus* as a rare medicinal fungus has attracted the attention of the scientific community.

*Inonotus obliquus* is the source of abundant natural products and has a lot of active components [8]. At present, in the analysis of *I. obliquus*, most of the bioactive substances are polysaccharides, triterpenes, and polyphenols [9,10]. There are also many studies on the efficacy of *I. obliquus* and the pharmacological effects of active compounds. Many pharmacological and pharmacodynamic studies have proved that *I. obliquus* has many functions, such as antitumor, antiviral, antioxidation, and anti-inflammatory activities, regulating blood glucose, regulating blood lipid, regulating blood pressure, improving immunity, and protecting the gastrointestinal tract and kidneys [9,10,11,12,13,14,15,16,17].

**Figure 1:**
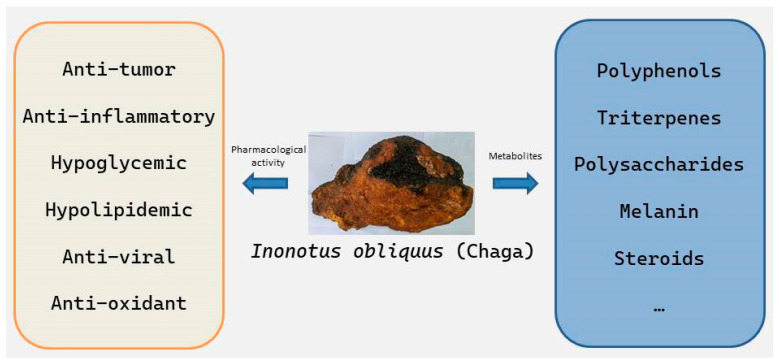
Natural products and health care functions of *Inonotus obliquus*.

Because of its various natural products and outstanding health care and pharmacological effects (Figure 1), it has attracted the attention of researchers. In previous reviews of *I. obliquus*, researchers have contributed excellent results, but most of the reviews focused on the pharmacological effects and active ingredients of *I. obliquus*, and many studies dating back more than ten years were cited. Therefore, this review focuses on the recent studies on *I. obliquus* and discusses the research progress on the regulation mechanism of *I. obliquus* metabolism on the basis of previous studies.

## 2. Materials and Methods

PubMed, Scopus, Web of Science, and Google Scholar were used to collect relevant studies. The search terms used were “Chaga”, “*Inonotus obliquus*”, “*Inonotus obliquus* metabolism”, “health benefits of *Inonotus obliquus*”, “*Inonotus obliquus* Anticancer”, “*Inonotus obliquus* antioxidant”, “*Inonotus obliquus* anti-diabetic”, “*Inonotus obliquus* anti-inflammatory”, “*Inonotus obliquusn* Antimicrobial”, and “review of *Inonotus obliquus*”. EndNote X9 and VOSviewer (version 1.6.20) were used to screen and collate references [18]. More recent articles were reviewed according to their publication years (most were within the last 5 years). The image was drawn using ChemSpider (http://www.chemspider.com/) and Microsoft Paint 3D software (6.2410.13017.0); the table was made using Microsoft Excel software (2010).

## 3. Main Active Components of *I. obliquus*

*Inonotus obliquus* lives in very cold areas and is subject to regular seasonal environmental pressure, including low temperature, ultraviolet radiation, and the invasion of pathogenic microorganisms [9,19]. In order to cope with various environmental stresses, *I. obliquus* has developed a series of defense mechanisms, which also lead to the production of many bioactive substances and the accumulation of a large number of metabolites [3,9]. These compounds have become the most important part of *I. obliquus* to play pharmacological roles.

### 3.1. Polysaccharides

Polysaccharides are a kind of polymeric carbohydrate composed of a glycosidic bond sugar chain and at least 10 monosaccharides [20]. Polysaccharides are not a pure chemical substance but a mixture of different degrees of polymerization [21]. Plants and fungi are important potential sources of polysaccharides [22,23,24]. Among all the active components of *I. obliquus*, polysaccharides have the most extensive biological activities, such as anticancer, hypoglycemic, anti-inflammatory, and antioxidant effects [25]. Hu et al. elucidated the molecular structure of a polysaccharide extracted from *I. obliquus* and revealed that it was mainly composed of a six-carbon pyarose Glc unit with an alpha configuration, with a major glycosidyl linkage at the (1 → 4) position, and mainly affected glycerophospholipid metabolism and other lipid metabolic pathways in mice [26]. Su et al. analyzed the monosaccharide composition of *I. obliquus* polysaccharides (IOPSs) with high-performance liquid chromatography, which were structured into mannose, rhamnose, glucose, galactose, xylose, and arabinose, and the molar ratio is 2.2:1.1:11.8:2.8:2.7:1.0. This study also confirms that *I. obliquus* polysaccharides have antitumor activity against osteosarcoma and have the potential for clinical application in osteosarcoma treatment [27]. Ding et al. isolated a novel polysaccharide, with a weight average molecular weight of 6886 Da was obtained from the black crystal region of *I. obliquus*, which contained mainly →4)-α-Glcp-(1→, α-Glcp-(1→, →6)-β-Glcp-(1→, →4, 6)-β-Glcp-(1→, →3)-α-Glcp-(1 → and →3, 6)-β-Glcp-(1→ residues and could inhibit pancreatic cancer cell proliferation and induce cell cycle arrest in AsPC-1 and SW1990 cells [28].

### 3.2. Triterpenes

Triterpenes are a kind of natural product with various biological activities, such as anticancer, anti-inflammatory, antioxidant, antiviral, antibacterial, and antifungal, etc., and can be isolated from plants, animals, or fungi [29]. Kim et al. fractionated the extract of *I. obliquus* with diaion HP-20 resin to obtain triterpenoid components and analyzed the composition of triterpenoids by HPLC and LC-ESI-MS, which were betulinic acid, betulin, trametenolic acid, inopodiol, and an unknown triterpenoid [30]. Sagayama et al. used the proliferation assay of human follicle dermal papilla cells to carry out bioassay-guided fractionations of the extract of *I. obliquus* to obtain lanostane-type triterpenes, then they verified that lanostane-type triterpenoids (1, 3, 4, and 5) could stimulate the growth of hair [31].

### 3.3. Polyphenols

The chemical characteristics of polyphenols are related to phenols and have strong antioxidant properties. Edible plants and fungi are increasingly recognized as sources for the recovery of bioactive polyphenols [32]. Since polyphenols, the predominant antioxidants in plants, are one of the most important theories to explain the aging process, these substances have attracted intense interest from researchers [33]. Lee et al. isolated inonoblins A (1), B (2), and C (3) as well as phelligridins D (4), E (5), and G (6) from the methanolic extract of *I. obliquus* and determined the structures of the compounds by extensive spectroscopic analysis [34]. In order to investigate the extraction process as well as antioxidant activity of polyphenols from *I. obliquus*, ten compounds were extracted under optimal conditions by Wang et al., namely procyanidin, caffeic acid, p-coumaric acid, isorhamnetin-3-o-glucoside, astilbin, tangeretin, gallic acid, kaempferol, quercetin, and catechin 7-xyloside. It can be seen that there is a large number of structurally different polyphenols in *I. obliquus* with great medicinal potential [35].

### 3.4. Others

The present study shows that *I. obliquus* also contains other bioactive chemical components. Burmasova et al. precipitated melanin from the aqueous extract of *I. obliquus* and the melanin fraction isolated by organic solvents. Analysis of melanin’s physical and chemical properties revealed the difference in particle size and charge, antioxidant properties, and redox potential between melanins and their fractions [36]. Zou et al. isolated six undescribed steroids from the fungus *I. obliquus* [37]. Niu et al. isolated and purified three water-soluble macromolecules from *I. obliquus*, which belong to lignin–carbohydrate complexes [2]. The content of natural products in different tissues of *I. obliquus* also varies significantly. The polyphenol content was higher in the epidermis than in internal tissue of every sclerotium; however, flavonoid and total triterpenoid content was lower in the epidermis of every sclerotium [38]. There will also be a distinction between epidermis and internal tissue for medicinal use.

**Figure 2:**
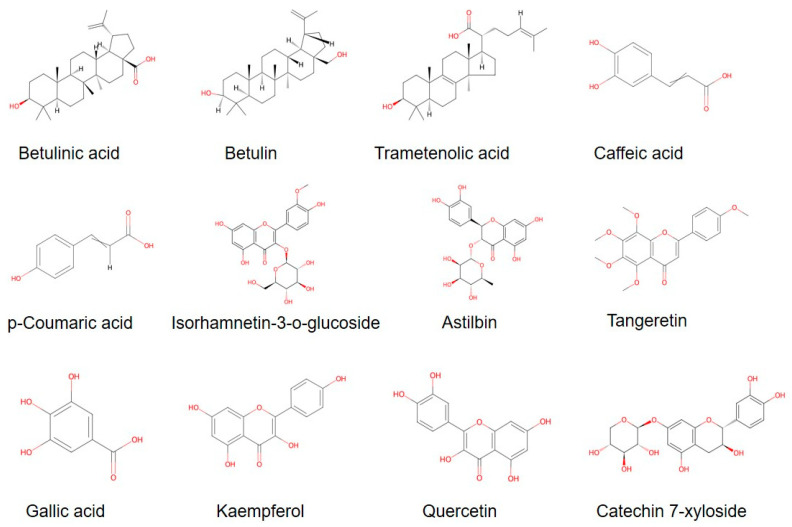
Some active components of *I. obliquus*.

There are very abundant natural product species in *I. obliquus*, and they have abundant activities and functions. We summarized some studies related to the active components of *I. obliquus* (Table 1, Figure 2). It is also a promising research direction to develop more efficient natural product extraction and isolation methods and obtain more bioactive substances from *I. obliquus*.

## 4. Health Care Functions of *I. obliquus*

As a rare medicinal fungus, *I. obliquus* has the beneficial effects of anti-inflammatory, antioxidation, antitumor, and antiviral activities, regulating blood sugar, enhancing immunity, improving gastrointestinal function, and so on. In recent years, many related animal experiments and clinical studies have been carried out, which are detailed below.

### 4.1. Antitumor Activity

*Inonotus obliquus* possesses the potential to reduce the incidence of tumorigenesis in healthy people by reducing tumor cell activity, inducing tumor cell apoptosis, and inhibiting the production of energy from tumor cells, thereby causing damage to tumor cells [39]. Su et al. extracted polysaccharide from *I. obliquus* and found that it could regulate osteosarcoma cell proliferation, migration, invasion, and apoptosis by inhibiting the activation of the Akt/mTOR signaling pathway [27]. Kim et al. evaluated the antiproliferative activity of methanol extract triterpenoid components against cancer cell lines, and the results showed that the triterpenoid fractions had dose-dependent anti proliferative activity on AGS, MCF-7, and PC3 cells; the effect was more significant at 250 and 300 μg/mL [30]. Fu et al. showed that inotodiol extracted from *I. obliquus* inhibited human cervical cancer cell lines HeLa cell migration and invasion via downregulating the levels of MMPs, and inotodiol could induce apoptosis by regulating the relationship of pro-apoptotic protein and anti-apoptotic protein [40]. Li et al. used a murine in vitro model to verify that IOPSs could promote the AOM/DSS-induced NLRP3 inflammasome activation and further verified the upregulation of ASC, caspase-1, and NLRP3 protein levels by IOPSs, which also enhanced the secretion of cytokine in tumor cells IL-1β and IL-18, thus illustrating a potential therapeutic role of IOPSs for colitis-associated cancer, and the effect of IOPSs is most obvious at high concentrations (150 μg/mL) [41].

### 4.2. Anti-Inflammatory Activity

Research has confirmed the anti-inflammatory effects of polysaccharides, polyphenols, and triterpenes from *I. obliquus*. Studies have shown that polysaccharides from *I. obliquus* reduce the release of pro-inflammatory cytokines in body cells by inhibiting the TLR4/NF-κB signaling pathway, thereby alleviating symptoms of endometritis induced by lipopolysaccharides (LPSs), and can serve as an effective drug for the prevention and treatment of LPS-induced endometritis. Sang et al. found that IOP downregulates the expression of TLR2 and TLR4 and inhibits the over-phosphorylation of NF-κB p65, the NF-κB signaling pathway inhibitor IκBα, and the MAPK signaling pathway components p38 and c-Jun N-terminal kinase (JNK), confirming that IOP is involved in the regulation of the NF-κB, p38, and JNK signaling pathways, thereby suppressing excessive inflammatory responses [42]. Inducible nitric oxide synthase (iNOS) is a key factor that may lead to excessive NO production and uncontrolled neuroinflammation. Therefore, inhibiting excessive NO production may be a new approach to controlling inflammation. Kou et al. demonstrated that inonotusols H–N can inhibit LPS-induced iNOS expression and that inonotusols I and L have strong interactions with iNOS protein, which may be beneficial for the treatment of neurodegenerative diseases [43]. Park et al. demonstrated that pure inotodiol and inotodiol-rich steroidal triterpene concentrates can reduce the expression of pro-inflammatory cytokines induced by ultraviolet (UV) and tumor necrosis factor (TNF-α), likely due to the inhibition of NF-κB signaling activation. These results illustrate the anti-inflammatory effects of inotodiol/inotodiol concentrates [44]. Yan et al. also demonstrated that IOP can inhibit the inflammatory response infected with T. gondii via regulating TLR2/TLR4-NF-κB/MAPKs pathways and exerting its anti-T. gondii role in vitro [11].

### 4.3. Hypoglycemic Effects

Diabetes, a disease with a high prevalence, has complex and diverse pathogenesis that is associated with both genetic factors and environmental factors such as age and lifestyle. Its long-term progression can lead to chronic damage to the eyes, heart, blood vessels, and nerves. Current antidiabetic drugs often have side effects such as hypoglycemia. Delgersaikhan N et al. induced hyperglycemia in experimental animals by destroying pancreatic β-cells with alloxan and found that water extract of *I. obliquus* (WEIO) could significantly restore body weight, reduce fasting blood glucose levels, improve glucose tolerance, and increase insulin levels, confirming that *I. obliquus* has strong hypoglycemic and pancreatic islet cell function-enhancing abilities [45]. Wang et al. found that the PI3K/Akt signaling pathway in diabetic mice is inhibited compared to normal mice, and the activation of this pathway is often accompanied by a reduction in insulin resistance, which improves insulin resistance and affirms the important role of PI3K/Akt in regulating glucose metabolism in type 2 diabetes (T2DM). IOPSs can significantly restore body and fat mass in diabetic mice after oral administration, reduce fasting blood glucose levels, improve glucose tolerance, increase hepatic glycogen levels, and ameliorate insulin resistance [46]. Feng et al. found that *I. obliquus* increases the levels of nitric oxide synthase (NOS) isoforms eNOS and nNOS to promote beneficial renal physiological effects through a cGMP-dependent pathway, reduces iNOS levels to mitigate pro-inflammatory responses, and inhibits phosphodiesterase 5 (PDE5) to decrease cGMP hydrolysis. The NOS-cGMP-PDE5 signaling pathway can be regulated to counteract high-fat diet (HFD)/streptozotocin (STZ)-induced disturbances in glucose and lipid metabolism and renal function [47]. Xu et al. also used streptozotocin (STZ) to induce diabetic model mice and demonstrated the excellent glucose-lowering effect of IOPSs through serum analysis [48].

### 4.4. Hypolipidemic Effects

*Inonotus obliquus* possesses certain cholesterol-lowering and blood-pressure-regulating effects. Ding et al. discovered that *I. obliquus* acidic polysaccharides (IOP-A) increase the expression of CYP7A1 and SR-B1 proteins, which regulate cholesterol transformation and transport in the livers of diet-induced hyperlipidemic rats, thereby reducing cholesterol levels [49]. Mo et al. demonstrated that IOP can reduce steatosis and inflammatory cells while stimulating the gene and protein expression of AMPK, SREBP-1C, FAS, and ACC in mouse livers, indicating that IOP has lipid-lowering effects both in vivo and in vitro and may hold potential for development in lipid-lowering applications [50].

### 4.5. Antiviral Activity

Polysaccharides from *I. obliquus* can inhibit the proliferation of viral cells within the body and prevent the binding of viral cells to target cells, exhibiting antiviral activity. Teplyakova et al. found that water extract of *I. obliquus* (WEIO) has high antiviral activity against SARS-CoV-2. Under optimal preparation conditions, the IC50 of WEIO for SARS-CoV-2 replication is 0.75 μg/mL, suggesting its potential use as a therapeutic and preventive agent [51]. In another study, the S1-carboxy-terminal domain of the SARS receptor binding CoV-2 domain was found to be closely associated with glucans, galactomannan, and betulinic acid. At TRP-436, ASN-437, and ASN-440 sites, the receptor-binding domain of the most recent SARS-CoV-2 isolate has a furin cleavage site that was not seen in previous isolates. Because this reaction interacts more frequently with ACE-2, the virus is able to infect more people, and *I. obliquus* may be used in combination with other drugs to combat SARS-CoV-2 [52]. Tian et al. determined the broad-spectrum antiviral activity of IOPSs, demonstrating that IOPSs effectively inhibited at least five different families, including RNA viruses (*Caliciviridae*, *Coronaviridae*, and *Orthomyxoviridae*) and DNA viruses (*Alphaherpesvirinae* and *Parvovirus*) [12]. At the same time, ribavirin was used as a control, and despite its more potent antiviral activity than IOPSs, in vitro toxicity assays showed that ribavirin exhibited higher toxicity than IOPSs.

### 4.6. Antioxidant Activity

In the study by Kou et al., the steroidal triterpenoid compound 2α-hydroxy-inotodiol (2α-HI, 1) was found to possess significant neuroprotective effects, significantly alleviating oxidative stress damage, reactive oxygen species (ROS) accumulation, and mitochondrial damage induced by H_2_O_2_ in SH-SY5Y cells. Additionally, they discovered that the neuroprotective effects of 2α-HI against H2O2-induced oxidative stress and apoptosis were mediated by the Nrf2 and BDNF/TrkB/ERK/CREB signaling pathways [53]. Li et al. identified phelligridin D from *I. obliquus*, which alleviates oxidative stress under high-glucose conditions by reducing reactive oxygen species (ROS) and malondialdehyde (MDA) levels while enhancing the activities of superoxide dismutase (SOD) and catalase (CAT) [53]. Phelligridin D also inhibits the secretion of transforming growth factor-β1 (TGF-β1) and downstream connective tissue growth factor (CTGF), thereby reducing the main components of the extracellular matrix (ECM), including collagen IV, fibronectin, and laminin. Moreover, under high-glucose conditions, phelligridin D activates nuclear factor erythroid 2-related factor 2 (Nrf2) in mesangial cells, contributing to its protective effects. These findings can be effectively applied in the medical field.

Pharmacological research on *I. obliquus* is extending from basic science to applied technology, and its multidisciplinary characteristics provide broad prospects for the development of natural drugs. Therefore, summarizing the recent basic research on *I. obliquus* will help to exert its broader value (Table 2).

## 5. Mechanism of Metabolite Synthesis

### 5.1. Polyphenols

The biosynthesis of hispidin polyphenols begins with the phenylpropanoid pathway, where phenylalanine ammonia lyase (PAL), cinnamic acid 4-hydroxylase (C4H), 4-coumarate-CoA ligase (4CL), and chalcone isomerase (CHI) catalyze the formation of hispidin precursors [54]. Zhou et al. found that UV irradiation inhibited the growth and biomass accumulation of *I. obliquus* but promoted the synthesis and secretion of polyphenols to resist UV stress, especially hispidin. They also observed that UV radiation upregulated the expression of metabolism-related genes and enhanced the activity of PAL and CHI in the phenylpropanoid pathway. PAL not only catalyzes the first step of the phenylpropanoid metabolic pathway but also regulates the entire pathway. p-Coumaroyl-CoA, catalyzed by C4H and 4CL, is converted into different metabolites by CHI. Enhanced enzyme activities in the phenylpropanoid pathway promote the accumulation of polyphenolic compounds in *I. obliquus* [55].

### 5.2. Triterpenes

Fungi synthesize triterpenes in the cytoplasm using acetyl-CoA as a substrate through the mevalonate (MVA) pathway, with squalene as a precursor that can be converted into its derivatives under the action of appropriate enzymes. In this process, 3-hydroxy-3-methylglutaryl-CoA reductase (HMGR), farnesyl pyrophosphate synthase (FPS), squalene synthase (SQS), and lanosterol synthase (LS) are key enzymes in this metabolic pathway [56]. Among these enzymes, farnesyl pyrophosphate synthase (FPS) (EC 2.5.1.10) is a critical branch point and major chain elongation enzyme, belonging to the e family of prenyltransferases [57]. Lin et al. investigated the effects of elicitors extracted from host and microbial sources, specifically birch bark (BB) and birch rhizosphere soil (BS), on the accumulation of triterpenes and FPS expression levels in *I. obliquus*. BB can increase triterpene accumulation to some extent, while BS has an inhibitory effect on triterpene accumulation, with significant differences in its inhibitory effects on BB [58].

### 5.3. Polysaccharides

Hua et al. used adaptive radiation-induced mutagenesis (ARTP) technology to mutate *I. obliquus* protoplasts to elucidate the biosynthetic pathways and functional genes related to high polysaccharide production [59]. The mutant genes associated with polysaccharide biosynthesis include those encoding glucose-1-phosphate uridylyltransferase, phosphoglucose isomerase, glycosidases, and glycosyltransferases. Among these enzymes, phosphoglucose isomerase (PGI) is involved in the pentose phosphate pathway, glycolysis/gluconeogenesis, and amino sugar and nucleotide sugar metabolism. Wang et al. studied the biological function of PGI in *Pleurotus ostreatus* and used RNA interference technology to construct a *P. ostreatus* polysaccharide gene-silenced strain. They found that the biomass of the *P. ostreatus* polysaccharide gene-silenced strain was significantly lower than that of the wild-type strain, with extracellular polysaccharide (EPS) and intracellular polysaccharide (IPS) levels increasing by 1.5–3 times and 1.5 times, respectively [60]. He et al. investigated the effects of carbon sources on polysaccharide biosynthesis and found that different carbon sources significantly affected the expression levels of key genes in the polysaccharide biosynthesis pathway, PGI and UDP-Glc4-epimase (UGE). Carbon sources differentially regulate the expression levels of polysaccharide biosynthesis-related genes, which in turn affect the synthesis, structure, and function of *I. obliquus* polysaccharides [61]. Lu et al. found that lignocellulosic materials significantly increased polysaccharide content and α-glucosidase inhibition rate and advanced the onset of α-glucosidase inhibitory activity, thereby promoting polysaccharide synthesis [62]. Wang et al. used elicitors such as VB6, VB1, betulin, and birch extract to regulate *I. obliquus*, which promoted EPS production and enhanced the inhibitory activity of polysaccharides against α-glucosidase [63].

**Figure 3:**
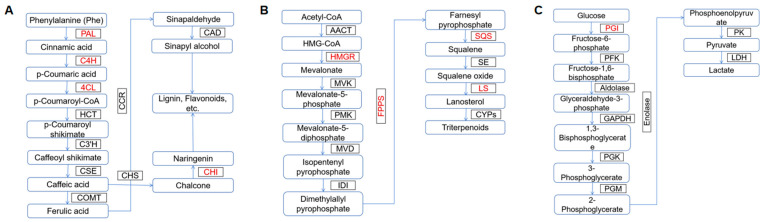
Main synthetic pathways of natural products from *I. obliquus*. (**A**) Phenylpropanoid biosynthesis pathway. Enzymes: PAL, phenylalanine ammonia lyase; C4H, cinnamic acid4 hydroxylase; CAD, (hydroxy)cinnamyl alcohol dehydrogenase; HCT, hydroxycinnamoyl-CoA:shikimate/quinate hydroxycinnamoyltransferase; C3′H, p-coumaroyl shikimate3′ hydroxylase; 4CL, 4-hydroxycinnamoyl-CoA ligase; CSE, caffeoyl shikimate esterase; CCR, cinnamoyl-CoA reductase; COMT, caffeic acid/5-hydroxyferulic acid O-methyltransferase; CAD, cinnamyl alcohol dehydrogenase; CHS, chalcone synthase; CHI, chalcone isomerase. (**B**) Polysaccharides biosynthesis pathway. Enzymes: PGI, phosphoglucose isomerase; PFK, phosphofructokinase; aldolase; GAPDH, glyceraldehyde-3-phosphate dehydrogenase; PGK, phosphoglycerate kinase; PGM, phosphoglycerate mutase; enolase; PK, pyruvate kinase; LDH, lactate dehydrogenase. (**C**) Triterpenes biosynthesis pathway. Enzymes: AACT, acetyl-CoA C-acetyltransferase; HMGR, HMG-CoA reductase; MVK, mevalonate kinase; PMK, phosphomevalonate kinase; MVD, mevalonate diphosphate decarboxylase; IDI, isopentenyl-diphosphate delta isomerase; FPPS, farnesyl diphosphate synthase; SQS, squalene synthase; SE, squalene epoxidase; LS, lanosterol synthase; CYPs, cytochromes P450 (the red font represents genes that have been studied so far).

Research on the synthesis mechanism of active ingredients in B. obliquus is not only the core link in the development of natural drugs but also a bridge connecting basic science and industrial application. Elucidation of the molecular mechanisms will lay the foundation for subsequent metabolic engineering optimization and synthetic biology development (Table 3, Figure 3). Through the intersection of multiple disciplines (such as genomics, metabolomics, and enzyme engineering), the whole chain of innovation from resource conservation to precision medicine is expected to be realized in the future.

## 6. Discussion

### 6.1. Pharmacological Effects and Diversity of Active Ingredients

As a traditional medicinal fungus, *I. obliquus* has been widely verified for its core pharmacological activity. Studies have shown that its antitumor, hypoglycemic, anti-inflammatory, and immunomodulatory effects are particularly prominent. For example, betulinoporol and triterpenoids exert anticancer effects by inhibiting tumor cell proliferation and inducing apoptosis, while polysaccharides such as β-glucans enhance immune function by activating macrophages and regulating the TLR4/NF-κB signaling pathway [11,42,43,44]. In addition, the hypoglycemic effect of *I. obliquus* is closely related to the glycoproteins and water-soluble polysaccharides in sclerotium, especially in non-insulin-dependent diabetes mellitus [62]. To investigate the metabolite richness in *I. obliquus*, metabolome studies have also been carried out. Many bioactive polyphenols including flavonoids and phenolic acids, such as caffeic acid, vanillic acid, isorhamnetin-3-O-arabinoside, and 3′,4′,7-trihydroxyflavone were found [64]. Notably, its polyphenolic components, such as melanin derivatives, exhibit anti-aging potential by scavenging free radicals and modulating antioxidant enzyme activities [65].

### 6.2. Production Technology Innovation and Scale Challenges

Traditional *I. obliquus* relies on wild collection, but artificial cultivation technology has made a breakthrough in recent years [66,67,68]. For example, by optimizing the strain and cultivation conditions, large-scale fruiting body production and increasing the yield of polysaccharides and triterpenoids were achieved [38,68,69]; endophytes and biotechnology were used to promote the growth and production of bioactive substances of *I. obliquus* [70]; supercritical CO_2_ extraction and fermentation are widely used for active ingredient extraction due to their high efficiency and environmental protection, while liquid submerged fermentation shorts the production cycle and increases the mycelium biomass [71]. However, artificial domestication still faces problems such as strain degradation and complex cultivation conditions, which require further optimization to improve its stability and repeatability.

### 6.3. Limitations in Clinical Application and Product Development

Although the pharmacological effects of *I. obliquus* are remarkable in animal models, clinical data are still insufficient, and in particular the synergistic effects and long-term safety of its components are not clear [9]. The pharmacological effects of *I. obliquus* were generally dose-dependent, and the effects of its core active ingredients (polysaccharides, triterpenes, polyphenols, etc.) were significantly enhanced in a specific concentration range. For example, effective concentrations for antitumor effects are in the microgram range (μg/mL), whereas doses for hypoglycemic and antioxidative effects are relatively low [65]. It is also important to determine any potential antagonistic or synergistic interactions of *I. obliquus* when taken with other drugs, and therefore pharmacologic and pharmacokinetic studies of *I. obliquus* as a therapeutic agent are also urgently needed. At present, Betula fuscoporitol capsules, energy drinks, and other products have been marketed, but their bioavailability is limited by their macromolecular structure (for example, polysaccharides need to be decomposed into short-chain fatty acids by intestinal flora) and metabolic pathways [72,73,74]. In addition, different extraction processes may lead to fluctuations in the active ingredient content and affect product consistency [75]. At the same time, it is necessary to combine metabolomics to analyze the metabolic pathways of active ingredients in vivo, develop nanocarriers technology to improve targeting and bioavailability, promote multi-center clinical trials, and establish a standardized component content and efficacy evaluation system [64,68,76,77,78]. Meanwhile, the metabolic pathways of *I. obliquus* can be optimized by gene editing to achieve efficient synthesis of bioactive components [79,80,81]. In addition to drugs, functional food and cosmetic raw materials can be developed to meet diverse health needs [82,83,84].

The research on *I. obliquus* has shifted from traditional medicinal experience to modern scientific verification. However, its industrialization still faces the challenges of technical bottleneck and insufficient clinical verification. In the future, it is necessary to integrate multidisciplinary technologies to break through the complexity of artificial cultivation and ingredient metabolism and promote the transformation from natural resources to high-value-added products.

## Figures and Tables

**Table 1:** Main active components of *I. obliquus*.

Main Active Components of *Inonotus obliquus*	Specific Ingredients	Functions	References
Polysaccharide	A six-carbon pyarose Glc unit with an alpha configuration with a major glycosidyl linkage at the (1 → 4) position	Glycerophospholipid metabolism and other lipid metabolic pathways	[26]
	Mannose, rhamnose, glucose, galactose, xylose, and arabinose	Antitumor activity against osteosarcoma	[27]
	Polysaccharide contained mainly →4)-α-Glcp-(1→, α-Glcp-(1→, →6)-β-Glcp-(1→, →4, 6)-β-Glcp-(1→, →3)-α-Glcp-(1 → and →3, 6)-β-Glcp-(1→	Inhibit pancreatic cancer cell proliferation and induce cell cycle arrest in AsPC-1 and SW1990 cells	[28]
Triterpenes	Betulinic acid, betulin, trametenolic acid, inopodiol, and an unknown triterpenoid	-	[30]
	Lanostane-type triterpenes	Stimulate the growth of hair	[31]
Polyphenols	Inonoblins A (1), B (2), and C (3) as well as phelligridins D (4), E (5), and G (6)	-	[34]
	Procyanidin, caffeic acid, p-coumaric acid, isorhamnetin-3-o-glucoside, astilbin, tangeretin, gallic acid, kaempferol, quercetin, and catechin 7-xyloside	Antioxidant activity	[35]
Others	Precipitated melanin	-	[36]
	Six undescribed steroids	-	[37]
	Three water-soluble macromolecules belong to lignin–carbohydrate complexes	-	[2]

**Table 2:** Health care functions of *I. obliquus*.

Health Care Functions of *Inonotus obliquus*	Related Components and Mechanisms	Specific Biological Activities	References
Antitumor activity	A six-carbon pyarose Glc unit with an alpha configuration with a major glycosidyl linkage at the (1 → 4) position; glycerophospholipid metabolism and other lipid metabolic pathways	To reduce the incidence of cancer in a healthy population	[39]
	Inhibit the activation of Akt/mTOR signaling pathway	Regulates the proliferation, migration, invasion and apoptosis of osteosarcoma cells	[27]
	Contains oleanolic acid, betulin, betulin, inopodiol (a triterpenoid compound), and an unknown triterpenoid compound	Triterpenoid components exhibit dose-dependent anti-proliferative activities against AGS, MCF-7, and PC3 cells	[30]
	Downregulate the level of matrix metalloproteinases (MMPs)	Inhibit the migration and invasion of human cervical cancer cell line HeLa cells and induce apoptosis by regulating the relationship between pro-apoptotic proteins and anti-apoptotic proteins	[40]
	Promote the activation of the NLRP3 inflammasome induced by AOM/DSS; upregulate the protein levels of ASC, caspase-1 and NLRP3; enhance the secretion of cytokines IL-1β and IL-18 in tumor cells	The potential therapeutic effect on inflammation-associated cancers	[41]
Anti-inflammatory Activity	Downregulate the expression of TLR2 and TLR4, inhibit the excessive phosphorylation of NF-κB p65, as well as IκBα (an inhibitor of the NF-κB signaling pathway) and the components of the mitogen-activated protein kinase (MAPK) signaling pathway, p38 and c-Jun N-terminal kinase (JNK)	Inhibit excessive inflammatory response	[42]
	Inhibit LPS-induced iNOS expression, and inonotusols I and L have strong interactions with the iNOS protein	Beneficial to the treatment of neurodegenerative diseases	[43]
	Reduce the expression of pro-inflammatory cytokines induced by ultraviolet (UV) and tumor necrosis factor (TNF-α), which may be due to its inhibition of the activation of the NF-κB signaling pathway	The anti-inflammatory effects of inotodiol and its concentrates are demonstrated	[44]
	Regulate the TLR2/TLR4-NF-κB/MAPKs pathway	Inhibit the inflammatory response caused by Toxoplasma gondii (T. gondii) infection and exert its anti-Toxoplasma effect in vitro	[11]
Hypoglycemic Effects	Restore body weight, reduce fasting blood glucose levels, improve glucose tolerance, and increase insulin levels	Powerful hypoglycemic ability and the function of enhancing islet cell function	[32]
	PI3K/Akt signaling pathway	Restore the body weight and fat mass of diabetic mice, reduce fasting blood glucose levels, improve glucose tolerance, increase hepatic glycogen levels, and ameliorate insulin resistance	[46]
	Increase the levels of endothelial nitric oxide synthase (eNOS) and neuronal nitric oxide synthase (nNOS) isoenzymes; reduce the level of inducible nitric oxide synthase (iNOS) to alleviate pro-inflammatory responses, and inhibit phosphodiesterase 5 (PDE5) to reduce the hydrolysis of cyclic guanosine monophosphate (cGMP); regulate the NOS-cGMP-PDE5 signaling pathway	Counteracting glucose and lipid metabolism as well as renal function disorders induced by high-fat diet (HFD)/streptozotocin (STZ)	[47]
	Ameliorating serum profiling	Reduce blood glucose	[48]
Hypolipidemic Effects	Increase the expression of CYP 7A1 and SR-B1 proteins	Lower cholesterol levels	[49]
	Stimulate the gene and protein expression of AMPK, SREBP-1C, FAS, and ACC	Reduce blood lipids	[50]
Antiviral Activity	Exhibit high antiviral activity against SARS-CoV-2	Therapeutic and preventive agents	[51]
	Chaga mushroom components, beta glycan, galactomannan, and betulinic acid exhibited strong binding interaction with the S1-carboxy-terminal domain of the receptor-binding domain of SARS-CoV-2, mainly at TRP-436, ASN-437, and ASN-440 sites	An effective natural antiviral	[52]
	A variety of virus activities were inhibited using the IOPSs	IOPSs effectively inhibited at least five different families, including RNA viruses (Caliciviridae, Coronaviridae, and orthomyxoviridae) and DNA viruses (alphaherpesviridae and parvoviruses)	[12]
Antioxidant Activity	Alleviate H_2_O_2_-induced oxidative stress injury, reactive oxygen species (ROS) accumulation, and mitochondrial damage in SH-SY5Y cells; Nrf2 and BDNF/TrkB/ERK/CREB signaling pathways	Neuroprotective effect	[53]
	Reduce the levels of reactive oxygen species (ROS) and malondialdehyde (MDA), while enhancing the activities of superoxide dismutase (SOD) and catalase (CAT)	Alleviate oxidative stress	[53]

**Table 3:** Mechanism of metabolite synthesis in *I. obliquus*.

Relevant Metabolism	Biosynthesis	Genes	References
Polyphenols	Phenylpropanoid pathway	Phenylalanine ammonia lyase (PAL), cinnamic acid 4-hydroxylase (C4H), 4-coumarate-CoA ligase (4CL), and chalcone isomerase (CHI) catalyze the formation of hispidin precursors	[54]
	Phenylpropanoid pathway	UV radiation upregulated the expression of metabolism-related genes and enhanced the activity of PAL and CHI in the phenylpropanoid pathway	[55]
Triterpenes	Mevalonate (MVA) pathway	3-hydroxy-3-methylglutaryl-CoA reductase (HMGR), farnesyl pyrophosphate synthase (FPS), squalene synthase (SQS), and lanosterol synthase (LS) are key enzymes in this metabolic pathway	[56]
	The effects of elicitors extracted from host and microbial sources, specifically birch bark (BB) and birch rhizosphere soil (BS), on the accumulation of triterpenes and FPS expression levels in *I. obliquus*	BB can increase triterpene accumulation to some extent, while BS has an inhibitory effect on triterpene accumulation, with significant differences in its inhibitory effects on BB	[58]
Polysaccharides	The mutant genes associated with polysaccharide biosynthesis include those encoding glucose-1-phosphate uridylyltransferase, phosphoglucose isomerase, glycosidases, and glycosyltransferases	Phosphoglucose isomerase (PGI) is involved in the pentose phosphate pathway, glycolysis/gluconeogenesis, and amino sugar and nucleotide sugar metabolism	[59]
	Different carbon sources significantly affected the expression levels of key genes in the polysaccharide biosynthesis pathway, PGI and UDP-Glc4-epimase (UGE)	Carbon sources differentially regulate the expression levels of polysaccharide biosynthesis-related genes, which in turn affect the synthesis, structure, and function of *I. obliquus* polysaccharides	[61]
	Lignocellulosic materials	Increased polysaccharide content and α-glucosidase inhibition rate and advanced the onset of α-glucosidase inhibitory activity, thereby promoting polysaccharide synthesis	[62]
	Used elicitors such as VB6, VB1, betulin, and birch extract to regulate Inonotus obliquus	Promoted EPS production and enhanced the inhibitory activity of polysaccharides against α-glucosidase	[63]

## Data Availability

Data are contained within the article.
